# Electrocutaneous stimulus setting for identification of the ascending nociceptive pathway

**DOI:** 10.1186/1471-2202-14-S1-P272

**Published:** 2013-07-08

**Authors:** Huan Yang, Jan R Buitenweg, Hil GE Meijer

**Affiliations:** 1 Department of Mathematics, Electrical Engineering, Computer Science and Mathematics, University of Twente, Enschede, Postbus 217, 7500 AE, the Netherlands

## Background

**Figure 1:**
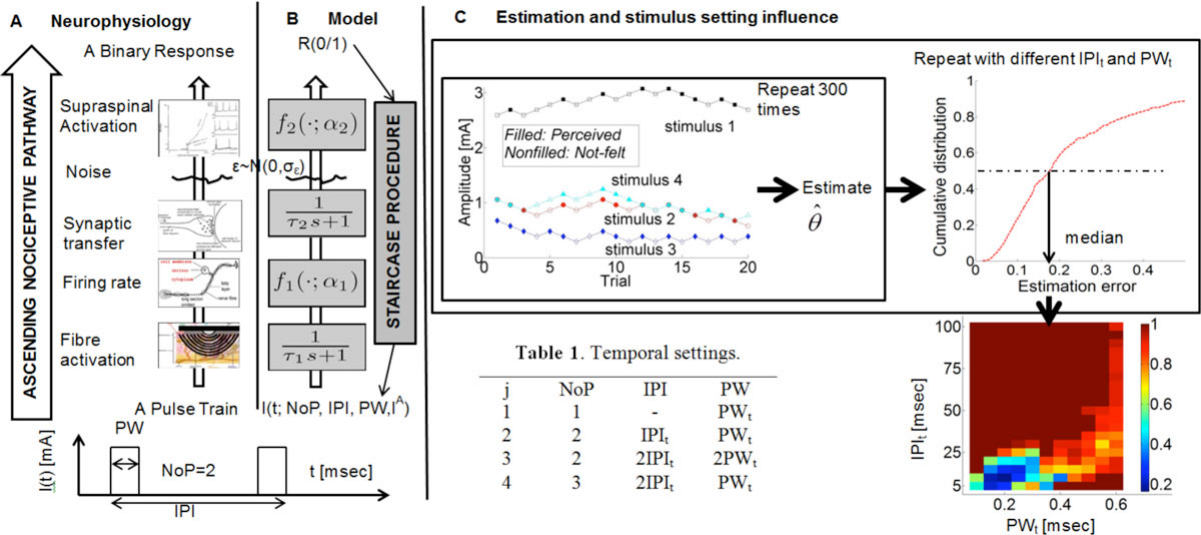
**A: Neurophysiology of the ascending pathway; B: Model; C: Estimation and stimulus setting influence**.

Malfunctioning of the ascending nociceptive pathway plays a key role in the development of chronic pain, e.g. central sensitization. In psychophysical experiments, subject's binary responses are measured during applying electrocutaneous stimulation. The stimulation delivers a square waveform parameterized by three temporal parameters, number of pulses (NoP), inter-pulse interval (IPI), pulse width (PW), and amplitude of the current (I^A^). These are the input-output measurements of this pathway accounting for peripheral activation, firing rate, synaptic transfer and supraspinal activation (Figure 1A). The relevant neurophysiological parameters may reflect the state of nociceptive system. To provide sufficient information to get the values of these parameters, multiple combinations of the settings in stimulus parameters are required. The challenge is to develop a reliable algorithm to estimate the parameters and to find an optimal stimulus setting.

## Methods

We model this pathway as a cascaded two leaky integrate-and-fire models followed by a nonlinear binary detector at the supraspinal level (Figure 1B). A white noise source presents at the output of neuronal activity. The model has five unknown parameters: two time constants (τ_1_, τ_2 _[msec]), two compound gain-threshold parameters (α_1_, α_2_) and standard deviation of the neuronal noise (σ_ε_). The multiple combinations of stimulus settings depend on two variables IPI_t _and PW_t _[msec] (Table 1). Using each setting to generate an artificial dataset, we estimate the parameters by maximizing the likelihood function of these parameters and the stimulus-response dataset. Varying IPI_t _and PW_t_, we look for an optimal setting of the stimulus.

## Results

For each setting, we present estimation errors in a cumulative distribution with particular values of the parameters, θ= (τ_1 _= 0.2, τ_2 _= 20, α_1 _= 0.1, α_2 _= 0.04, σ_ε _= 0.005). The chance with an estimation error below 17% is a half when IPI_t _is 10 and PW_t _is 0.2. Furthermore, we show the median of the estimation error in a 2D plot with varying IPI_t _and PW_t_.

## Conclusions

To identify the ascending nociceptive pathway, we have proposed an estimation approach using stimulus-response measurements. Optimal temporal settings of stimulus are found for a reliable estimation within the region of IPI_t _10-25 [msec] and PW_t _0.15-0.4 [msec].

